# A single injection of crystallizable fragment domain–modified antibodies elicits durable protection from SHIV infection

**DOI:** 10.1038/s41591-018-0001-2

**Published:** 2018-04-16

**Authors:** Rajeev Gautam, Yoshiaki Nishimura, Natalie Gaughan, Anna Gazumyan, Till Schoofs, Alicia Buckler-White, Michael S. Seaman, Bruce J. Swihart, Dean A. Follmann, Michel C. Nussenzweig, Malcolm A. Martin

**Affiliations:** 1 Laboratory of Molecular Microbiology, National Institute of Allergy and Infectious Diseases, National Institutes of Health, Bethesda, MD USA; 2 Laboratory of Molecular Immunology, Rockefeller University, New York, NY USA; 3 Center for Virology and Vaccine Research, Beth Israel Deaconess Medical Center, Boston, MA USA; 4 Biostatistics Research Branch, Division of Clinical Research, National Institute of Allergy and Infectious Diseases, National Institutes of Health, Bethesda, MD USA; 5 Howard Hughes Medical Institute, Rockefeller University, New York, NY USA

## Abstract

In the absence of an effective and safe vaccine against HIV-1, the administration of broadly neutralizing antibodies (bNAbs) represents a logical alternative approach to prevent virus transmission. Here, we introduced two mutations encoding amino acid substitutions (M428L and N434S, collectively referred to as ‘LS’) into the genes encoding the crystallizable fragment domains of the highly potent HIV-specific 3BNC117 and 10-1074 bNAbs to increase their half-lives and evaluated their efficacy in blocking infection following repeated low-dose mucosal challenges of rhesus macaques (*Macaca mulatta*) with the tier 2 SHIV_AD8-EO_. A single intravenous infusion of 10-1074-LS monoclonal antibodies markedly delayed virus acquisition for 18 to 37 weeks (median, 27 weeks), whereas the protective effect of the 3BNC117-LS bNAb was more modest (provided protection for 11–23 weeks; median, 17 weeks). Serum concentrations of the 10-1074-LS monoclonal antibody gradually declined and became undetectable in all recipients between weeks 26 and 41, whereas the 3BNC117-LS bNAb exhibited a shorter half-life. To model immunoprophylaxis against genetically diverse and/or neutralization-resistant HIV-1 strains, a combination of the 3BNC117-LS plus 10-1074-LS monoclonal antibodies was injected into macaques via the more clinically relevant subcutaneous route. Even though the administered mixture contained an amount of each bNAb that was nearly threefold less than the quantity of the single monoclonal antibody in the intravenous injections, the monoclonal antibody combination still protected macaques for a median of 20 weeks. The extended period of protection observed in macaques for the 3BNC117-LS plus 10-1074-LS combination could translate into an effective semiannual or annual immunoprophylaxis regimen for preventing HIV-1 infections in humans.

## Abstract (web summary)

Long-lived antibodies that can prevent viral infection of monkeys for 6 months may be a future alternative to an HIV vaccine.

## Main

Because an effective anti-HIV-1 vaccine is not currently available nor imminent, new approaches are needed to prevent HIV transmission. Such new strategies have included the use of bNAbs, isolated from infected persons with high titers of anti-HIV-1 neutralizing activity^1–3^. bNAbs are capable of neutralizing most circulating strains, targeting different nonoverlapping epitopes on the HIV-1 envelope spike, such as the CD4-binding site^3–5^, variable loop 1 and 2 (V1V2 loop)^2,6^, V3 loop^1,7,8^, the membrane proximal region^9^ and a series of epitopes spanning the gp120–gp41 interacting region^10,11^. Several bNAbs, including 3BNC117, VRC01, PGT121 and 10-1074, can protect macaques from simian–HIV (SHIV) infections^12–17^. In addition, these antibodies have been reported to control virus replication in chronically SHIV-infected monkeys^18–21^. Human studies using the VRC01 or 3BNC117 monoclonal antibodies, which target the CD4-binding site, or the 10-1074 monoclonal antibody, which binds to the base of the gp120 V3 loop and surrounding glycans, have shown that the antibodies are generally safe and active in vivo^22–25^. bNAb administration transiently reduces plasma viremia and delays rebound during treatment interruption in individuals with an HIV-1 infection^22–27^.

We previously reported that single intravenous (i.v.) injections of native VRC01, 3BNC117 or 10-1074 bNAbs (20 mg per kg body weight) prevented virus acquisition in macaques following repeated low-dose (RLD) challenges with tier 2 SHIV_AD8-EO_ as compared to control monkeys that received no anti-HIV-1 neutralizing monoclonal antibodies^12^. In that study, the 3BNC117 and 10-1074 bNAbs protected monkeys for a median of 13 and 12.5 weeks, respectively, whereas VRC01, possessing lower neutralizing activity against SHIV_AD8-EO_, blocked infection for a shorter period of time (a median of 8 weeks). In addition, the VRC01 monoclonal antibody, carrying a two-amino-acid substitution (LS) introduced into its crystallizable fragment domain that increased its serum half-life by two- to threefold^12,28^, was also evaluated. As compared to the unmodified VRC01, the VRC01 monoclonal antibody with the LS substitution (VRC01-LS) exhibited a longer median protective effect (14.5 versus 8.0 weeks).

Here we have examined two aspects of anti-HIV-1 immunoprophylaxis: (1) the long-term efficacy of the more potent 3BNC117 or 10-1074 bNAbs with the LS substitution in the crystallizable fragment infused individually through the i.v. route; and (2) the prevention of virus acquisition via the combination of LS-mutant 3BNC117 and 10-1074 monoclonal antibodies administered subcutaneously (s.c.). Our results show that a single infusion of the 10-1074-LS monoclonal antibody protected four of six monkeys challenged on a weekly basis for more than 6 months. In addition and despite volume limitations (1.0 ml), s.c. combination immunoprophylaxis conferred protection in five of six monkeys against RLD virus challenge for a median of 20 weeks.

## Results

### Neutralizing potency of the LS-modified monoclonal antibodies

**Fig. 1:**
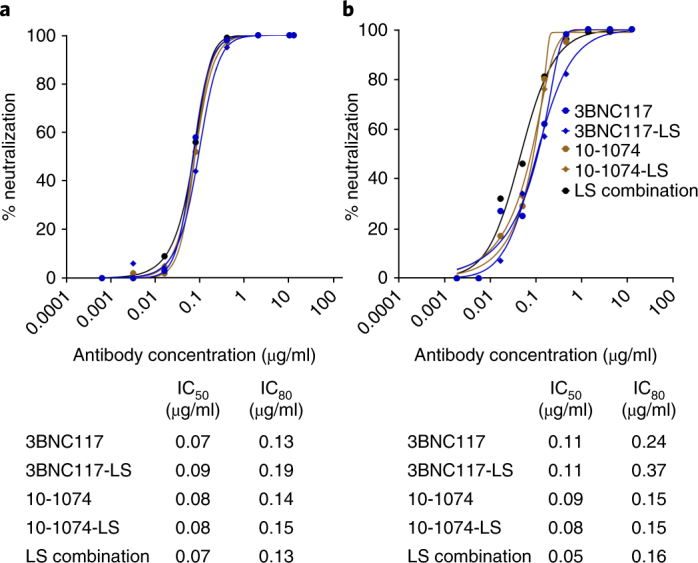
Neutralization sensitivity of broadly acting neutralizing anti-HIV-1 monoclonal antibodies against SHIV_AD8-EO_. **a**, Top, neutralizing activity of the indicated bNAbs was determined against SHIV_AD8-EO_ pseudovirions using TZM-bl target cells. Bottom, the calculated IC_50_ and IC_80_ values for the antibodies. **b**, Top, neutralizing activity of the indicated bNAbs was determined against replication-competent SHIV_AD8-EO_ in a single-round TZM-bl infectivity assay in the presence of indinavir. Bottom, the calculated IC_50_ and IC_80_ values for the antibodies.  The neutralization assays were repeated three times with similar results.

To examine the anti-SHIV_AD8-EO_ neutralizing activity of the native^12^ and LS-modified forms of 3BNC117 and 10-1074, we performed virus neutralization assays using either pseudotyped (Fig. 1a) or replication-competent (Fig. 1b) viruses during infections of TZM-bl cells. The half-maximal inhibitory concentrations (IC_50_s) of the native and LS-modified forms of the 3BNC117 and 10-1074 monoclonal antibodies were nearly indistinguishable in the TZM-bl pseudovirus assay (0.07 versus 0.09 µg/ml and 0.08 versus 0.08 µg/ml, respectively). Similarly, assays using replication-competent SHIV_AD8-EO_ showed IC_50_ values for the native and LS-modified forms of 3BNC117 and 10-1074 of 0.11 versus 0.11 µg/ml and 0.09 versus 0.08 µg/ml, respectively. The corresponding 80% inhibitory concentration (IC_80_) values were 0.24 versus 0.37 µg/ml and 0.15 versus 0.15 µg/ml for native and LS-modified forms of 3BNC117 and 10-1074 monoclonal antibodies, respectively. We conclude that the LS-modified forms of 3BNC117 and 10-1074 have neutralization activities similar to those of the native antibodies in these in vitro assays.

### 3BNC117-LS or 10-1074-LS monoclonal antibody administration confers long-term protection against repeated mucosal SHIV challenges

**Fig. 2:**
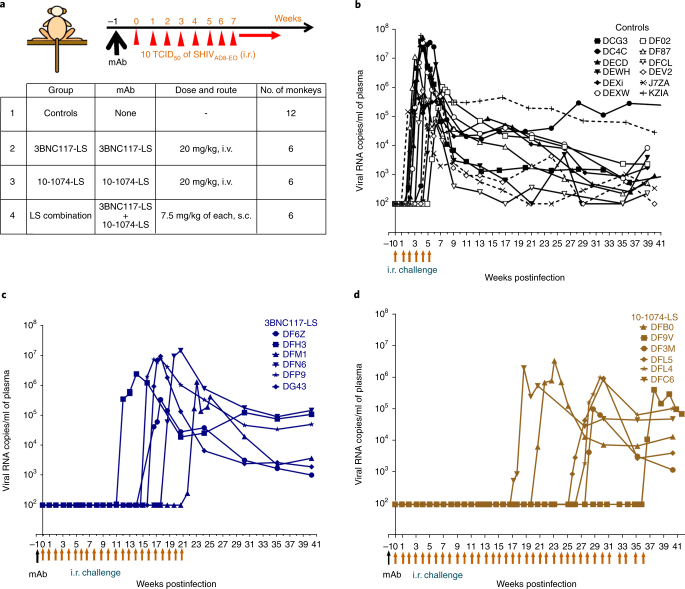
Crystallizable fragment domain–modified HIV monoclonal antibodies confer durable protection against repeated low-dose IR SHIV_AD8-EO_ challenges. **a**, Experimental design for assessment of the protective efficacy of monoclonal antibodies in rhesus macaques. Single doses of the indicated individual monoclonal antibodies (20 mg per kg body weight) or combination monoclonal antibodies (7.5 mg per kg body weight of each monoclonal antibody) were administered either i.v. or s.c. Macaques were challenged with SHIV_AD8-EO_ via the i.r. route weekly, beginning 1 week following monoclonal antibody (mAb) infusion. **b**, Plasma viral loads in rhesus macaques receiving no monoclonal antibody (controls; *n* = 12) challenged weekly with SHIV_AD8-EO_. **c**,**d**, Plasma viral loads in rhesus macaques (*n* = 6 per group) challenged weekly with SHIV_AD8-EO_ beginning 1 week after i.v. administration of 3BNC117-LS (**c**) or 10-1074-LS (**d**) monoclonal antibody.

To determine the protective efficacy of 3BNC117-LS and 10-1074-LS monoclonal antibodies in macaques, we performed intrarectal (i.r.) RLD challenge experiments. All monkeys were inoculated with 10 tissue culture infectious dose 50 (TCID_50_) of SHIV_AD8-EO_ at weekly intervals until they became viremic, as determined through real-time RT-PCR analysis (Fig. 2a). This inoculum size was previously shown to be equivalent to 0.27 animal infectious dose 50 (AID_50_)^12^. Twelve control monkeys, which received no monoclonal antibodies, became infected after two to six challenges, with a median of three weekly virus exposures needed to infect all 12 monkeys (Fig. 2b). The protective efficacy of 3BNC117-LS or 10-1074-LS was assessed following a single i.v. infusion of each monoclonal antibody (20 mg per kg body weight) in six monkeys. The macaques were challenged beginning one week after bNAb administration, and in addition to levels of viral RNA, we measured serum bNAb concentrations, anti-SHIV neutralizing titers and anti-bNAb responses.

**Fig. 3:**
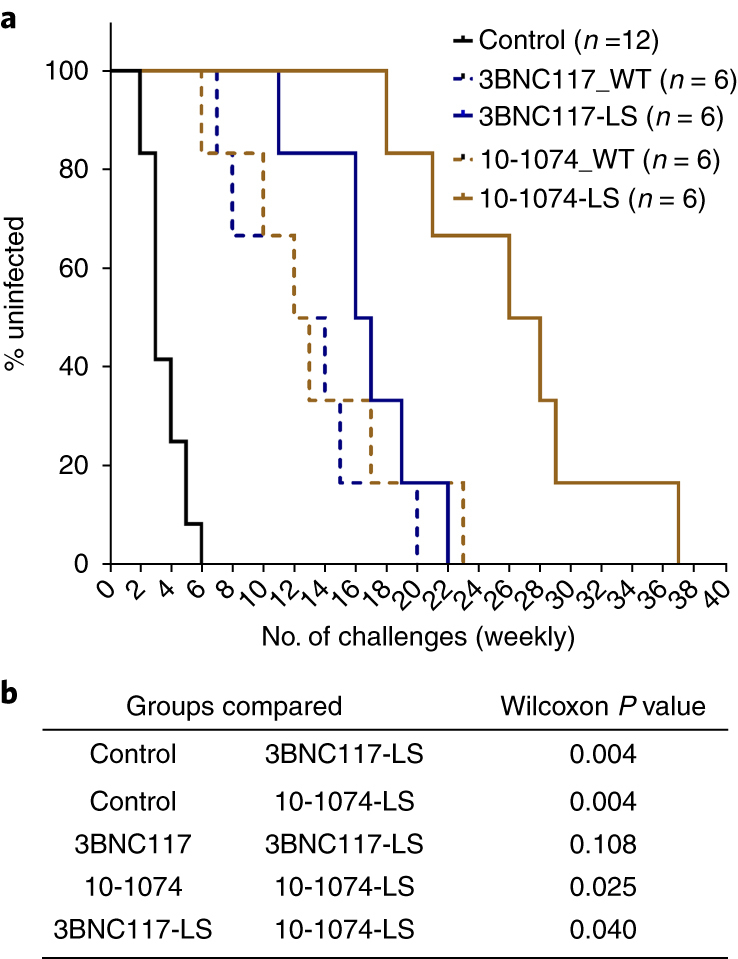
Protective effects of 3BNC117-LS and 10-1074-LS monoclonal antibodies against virus acquisition in rhesus macaques. **a**, Kaplan–Meier analysis was used to assess infection rates for controls and recipients of 3BNC117-LS and 10-1074-LS monoclonal antibodies and their native forms. The percentage of uninfected rhesus macaques following SHIV_AD8-EO_ i.r. challenge was assessed for the monoclonal antibody recipients (*n* = 6 per group) and control monkeys (*n* = 12). **b**, *P* values were determined using Wilcoxon rank-sum test (two-sided) comparing the number of challenges resulting in infections of control monkeys versus the individual monoclonal antibody–recipient group or between different monoclonal antibody–recipient groups.

The LS-modified bNAbs were well tolerated in all 12 monkeys. In the six 3BNC117-LS bNAb recipients, 11 to 23 challenges were required to establish infection, and the median time to virus acquisition was 17 weeks for this group of monkeys (Fig. 2c). In the case of the 10-1074-LS recipients, 18 to 37 virus challenges were needed to establish an infection, and the median time to virus acquisition was 27 weeks (Fig. 2d). The median times to virus acquisition in the recipients of the native 10-1074 and 3BNC117 monoclonal antibodies were 12.5 and 13 weeks, respectively^12^. Thus, the 10-1074-LS bNAb conferred a 2.2-fold increase (12.5 to 27) in the number of challenges needed to establish an infection compared with the unmodified 10-1074 monoclonal antibody, whereas the 3BNC117-LS bNAb conferred a modest 1.3-fold (13 to 17 challenges) improvement. The protective effects of the 3BNC117-LS and 10-1074-LS bNAbs were also compared to the control cohort using Kaplan–Meier analysis, in which the percentage of macaques remaining uninfected was plotted against the number of SHIV_AD8-EO_ challenges (Fig. 3a). As indicated in Fig. 3b, the recipients of 3BNC117-LS and 10-1074-LS monoclonal antibodies were significantly more resistant to SHIV_AD8-EO_ acquisition than the control monkeys (*P* = 0.004 and 0.004, respectively). Furthermore, 10-1074-LS was significantly different from native 10-1074 (*P* = 0.026), whereas the 3BNC117-LS was not statistically different from its native form (*P* = 0.108).

### In vivo protective activity is dependent upon monoclonal antibody pharmacokinetics

**Fig. 4:**
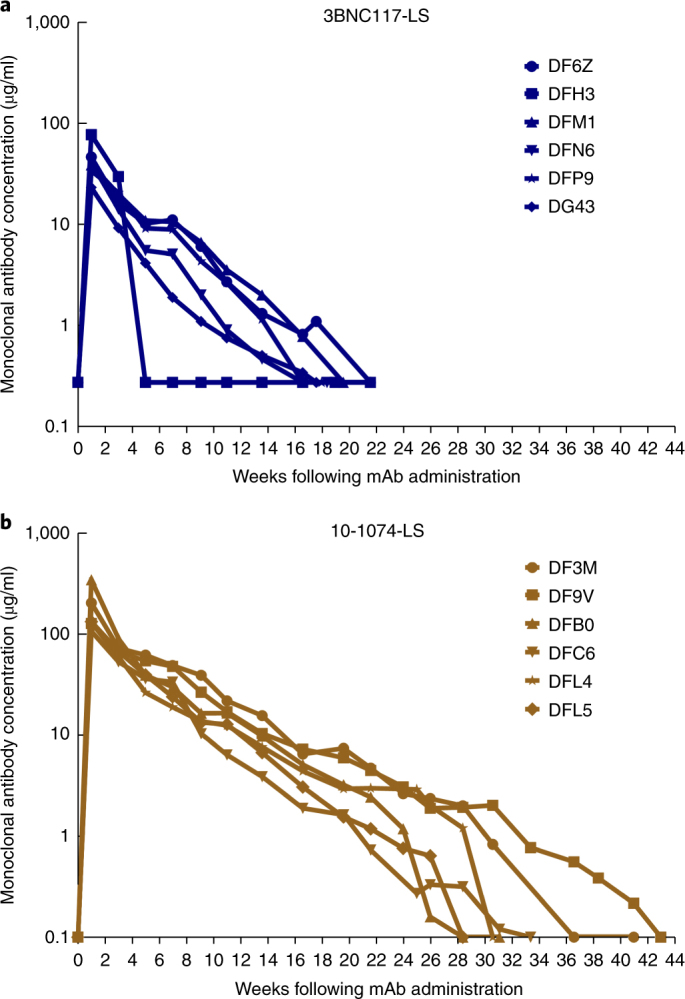
Serum antibody concentrations in rhesus macaques infused with crystallizable fragment domain–modified monoclonal antibodies. **a**, Concentrations of 3BNC117-LS antibody were measured in serum over the course of 6 months following a single i.v. infusion (20 mg per kg body weight) of the 3BNC117-LS monoclonal antibody using the TZM-bl cell assay. **b**, Concentrations of 10-1074-LS antibody were measured in serum for 9 months after i.v. infusion of a single 20 mg per kg body weight dose of 10-1074-LS monoclonal antibody using a TZM-bl cell assay. The assay was performed twice.

To determine how LS-modified bNAbs in monkey sera relate to protection, we measured the concentrations of these antibodies at various times after administration. The serum concentrations of 3BNC117-LS in five of six recipients gradually declined and became undetectable between weeks 16 and 22 following infusion (Fig. 4a and Supplementary Table 1). One 3BNC117-LS monoclonal antibody recipient (DFH3), however, experienced rapid decay of the administered antibody, and the monoclonal antibody concentrations in this monkey declined to undetectable levels by week 5 postinfusion. In contrast, none of the six macaques infused with the 10-1074-LS bNAb exhibited a rapid loss of the administered monoclonal antibodies; bNAbs were measurable until weeks 26–41 in this cohort of monkeys (Fig. 4b and Supplementary Table 1). The median serum-neutralizing activities of the 3BNC117-LS and 10-1074-LS monoclonal antibodies 1 week after infusion were 1:2,538 and 1:9,840, respectively (*P* = 0.0022, Wilcoxon rank-sum test; Supplementary Fig. 1). These values are similar to comparable titers (1:3,248 and 1:7,163) measured at 1 week following infusion of the native 3BNC117 and 10-1074 bNAbs, respectively^12^. Thus, despite exhibiting similar neutralization activities against SHIV_AD8-EO_ in vitro (Fig. 1), the neutralization titers of each bNAb at 1 week following infusion were different from one another in vivo, with higher titers measured for native 10-1074 and 10-1074-LS compared to native 3BNC117 and 3BNC117-LS.

The introduction of the LS-encoding mutations into the gene encoding VRC01 extended its serum half-life by two- to threefold^12,28^. The half-life of 3BNC117-LS ranged from 1.5 to 3.2 weeks (median, 2.8 weeks), whereas native 3BNC117 had a half-life of 0.7 to 1.7 weeks (median, 1.4 weeks), as shown in Supplementary Table 2. Similarly, the half-life of 10-1074-LS ranged from 3.0 to 4.9 weeks (median, 3.8 weeks) and the native monoclonal antibody exhibited a half-life of 0.6 to 3.2 weeks (median, 1.0 week), as shown in Supplementary Table 2. Thus, the LS-encoding mutation increased the half-lives of native 3BNC117 and 10-1074 by 2.0- and 3.8-fold, respectively (*P* = 0.0044 for 3BNC117-LS versus 3BNC117 and *P* = 0.0014 for 10-1074-LS versus 10-1074).

### bNAb combination immunoprophylaxis prevents SHIV acquisition

**Fig. 5:**
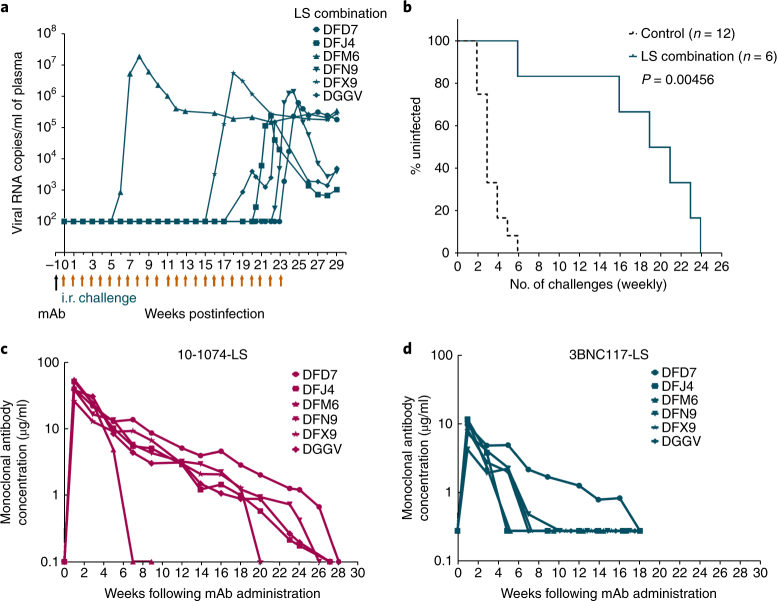
Protection efficacy of combination 3BNC117-LS plus 10-1074-LS monoclonal antibody administered s.c. to rhesus macaques. **a**, Plasma viral loads in rhesus macaques (*n* = 6) challenged repeatedly with SHIV_AD8-EO_ beginning 1 week after the s.c. administration of a single dose of the 3BNC117-LS plus 10-1074-LS monoclonal antibody mixture (7.5 mg per kg body weight of each). **b**, Kaplan–Meier survival curves show the percentage of rhesus macaques remaining uninfected following repeated SHIV_AD8-EO_ i.r. challenges required to establish infection of monoclonal antibody–combination recipients (*n* = 6) or controls (*n* = 12). *P* values were determined using the Wilcoxon rank-sum test (two-sided) comparing the number of challenges resulting in infection of the controls and monoclonal antibody–combination recipients. **c**,**d**, Concentrations of 10-1074-LS and 3BNC117-LS monoclonal antibodies were measured, using the TZM-bl cell assay, in serum of rhesus macaques administered a single injection of the monoclonal antibody mixture s.c. Antibody concentrations were measured twice.

In view of the extraordinary genetic diversity of HIV-1, it is likely that prophylaxis against HIV-1 in humans will require a combination of bNAbs targeting different epitopes on the viral envelope. In addition, i.v. antibody administration is far less desirable as a means of prophylaxis in humans than injection via the s.c. route. To evaluate the protective efficacy of combining bNAbs, we administered 3BNC117-LS plus 10-1074-LS monoclonal antibodies (7.5 mg per kg body weight of each) in a clinically relevant volume of 1 ml s.c. to a cohort of six macaques, which were challenged weekly with 10 TCID_50_ of SHIV_AD8-EO_, as described above. This monoclonal antibody dose was nearly three times lower than that used when each bNAb was i.v. administered individually (20 mg per kg body weight of each), as described earlier. As shown in Fig. 5a, combination monoclonal antibody prophylaxis conferred protection for 15 to 24 challenges in five of the six recipients. One monkey (DFM6) became infected after only six i.r. challenges. The median time to virus acquisition for the entire cohort was 20 weeks (*P* = 0.004 compared to untreated controls, Wilcoxon rank-sum test; Fig. 5b).

The concentrations of the 3BNC117-LS and 10-1074-LS monoclonal antibodies in sera from individual macaques were determined longitudinally (Fig. 5c,d). The serum concentrations of 3BNC117-LS monoclonal antibody declined to undetectable levels between 5 and 9 weeks in all but one of the six recipients (Fig. 5d and Supplementary Table 3). The sixth monkey, DFD7, maintained measurable concentrations of the 3BNC117-LS monoclonal antibody until week 18 following s.c. administration. In contrast, the 10-1074-LS bNAb gradually declined in five of the six monkeys and was detected in circulation until weeks 18–27 post administration in all five of these monkeys (Fig. 5c and Supplementary Table 3). One macaque (DFM6) experienced rapid loss of both monoclonal antibodies and became infected after six weekly challenges (Fig. 5a). The pattern of anti-HIV-1 serum neutralizing titers in recipients of the 3BNC117-LS plus 10-1074-LS monoclonal antibody mixture paralleled the serum concentrations of the 10-1074-LS monoclonal antibody, but not that of the 3BNC117-LS monoclonal antibody, in this macaque cohort (compare Supplementary Fig. 2 with Fig. 5c,d). Taken together, these results show that the 10-1074-LS monoclonal antibody was solely responsible for the protection of five of the six macaques after week 7.

### Monoclonal antibody concentration and neutralization activity predict the probability of infection

**Fig. 6:**
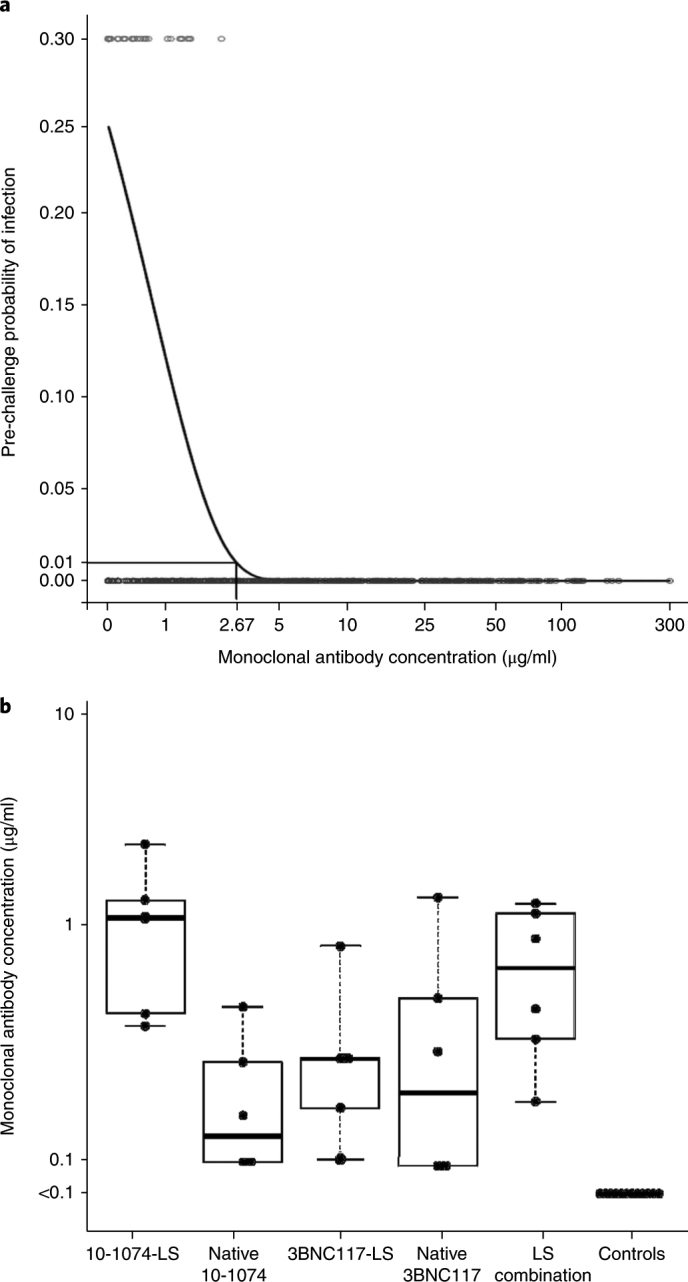
Antibody concentration predicts the probability of infection. **a**, Probit regression was used to model the probability of infection based on antibody concentrations in serum of rhesus macaques at the time of each SHIV_AD8-EO_ challenge. The probability of infection for the control monkeys (*n* = 12) was estimated to be 0.27. The fitted probability, determined using the probit regression model, is plotted for all recipients of LS-modified monoclonal antibodies (*n* = 18) and native monoclonal antibodies (*n* = 12; previously reported^12^). Each red circle indicates the monoclonal antibody concentration at the time of virus challenge resulting in infection; blue circles indicate the monoclonal antibody concentration at the time of virus challenge not resulting in infection. A monoclonal antibody concentration of 2.67 μg/ml in serum was predicted to have a 0.01 per-challenge infection probability. **b**, Concentrations of monoclonal antibodies in sera from rhesus macaques at the time of virus acquisition. The tops and bottoms of each box represent 75th and 25th percentiles, respectively. The bars above and/or below each box (whiskers) represent the entire spread of the data points, and the heavier line represents the median value for each group (*n* = 6 per group, except for controls, where *n* = 12). Some of the data points on the box plots are superimposed.

We used probit regression analysis to estimate an S-shaped curve that describes the per-challenge probability of infection as a function of the serum monoclonal antibody concentration (Supplementary Tables 1 and 3). The estimated probit curve for these studies is shown in Fig. 6a. Likelihood ratio tests indicated that the same probit curve could be applied for each of the following monoclonal antibodies: native 3BNC117 and 3BNC117-LS; native 10-1074 and 10-1074-LS; and the 3BNC117-LS plus 10-1074-LS combination. Thus, a given amount of monoclonal antibody from any of the five types evaluated predicted the same per-challenge infection probability. The serum monoclonal antibody concentration, corresponding to a per-challenge infection probability of 1%, was calculated to be 2.67 μg/ml (95% confidence interval, 1.85–3.48 for all of the monoclonal antibodies evaluated). As shown in Fig. 6b, the median monoclonal antibody serum concentrations at breakthrough of infection were calculated to be 0.13 and 1.07 μg/ml for native 10-1074 and 10-1074-LS, respectively; 0.20 and 0.28 μg/ml for native 3BNC117 and 3BNC117-LS, respectively; and 0.67 μg/ml for the bNAb combination. The slightly higher breakthrough plasma bNAb concentration observed for the 10-1074-LS monoclonal antibody (1.07 μg/ml) compared to the three other individual bNAbs analyzed in Fig. 6b cannot be presently explained despite the similar potencies of these antibodies when measured in vitro (Fig. 1). Perhaps this difference reflects currently unknown variables affecting bNAb activity in vivo that are not operative in vitro.

The protective efficacy of different serum antibody concentrations, relative to the per-challenge risk of infection, was also determined. Antibody efficacy was defined as 100% × ((1 – (per-challenge infection probability at a given antibody concentration)) / (per-challenge infection probability with no antibody)) (see statistical analysis in the Methods for details). The antibody efficacies were 97% (95% confidence interval, 92–100%) and 61% (95% confidence interval, 49–77%) for all of the wild-type and crystallizable fragment domain–modified 3BNC117 and 10-1074 bNAbs used in this study at serum concentrations of 3.0 and 1.0 μg/ml, respectively.

### Anti-antibody responses against human monoclonal antibodies can cause their rapid decay in macaques

Macaque recipients of human anti-HIV-1 bNAbs variably produce anti-antibodies that are associated with accelerated bNAb clearance, and in prevention experiments, this leads to virus acquisition^12^. In the current study, one monkey (DFH3), in the cohort of six 3BNC117-LS monoclonal antibody recipients, developed anti-antibodies at 3 to 4 weeks after infusion and experienced a decline of bNAb concentrations to undetectable levels by week 5 (Fig. 4a and Supplementary Fig. 3a). The most striking example of anti-antibody production occurred in the bNAb combination experiment, where four of six monkeys developed specific anti-3BNC117-LS antibodies after 4 to 6 weeks, and one of these macaques (DFM6) also produced anti-antibodies to the 10-1074-LS component of the mixture in a similar time frame (Supplementary Fig. 3c,d). As expected, the generation of anti-antibodies against both 3BNC117-LS and 10-1074-LS monoclonal antibodies in monkey DFM6 was associated with a rapid decline of both serum bNAb concentrations (Fig. 5c,d) and measurable neutralization titers in this animal (Supplementary Fig. 2), resulting in the establishment of infection.

## Discussion

The most striking result obtained in this study is the long period of protective efficacy conferred by a single injection of crystallizable fragment domain–modified human anti-HIV-1 neutralizing antibodies in macaques compared to that previously reported^12^. A single i.v. infusion of the 10-1074-LS bNAb protected a cohort of six monkeys for up to 8.5 months (18–37 weeks). The introduction of the LS substitution into 10-1074 lengthened the median time until SHIV_AD8-EO_ acquisition from 12.5 to 27 weeks. The administered 10-1074-LS bNAb was measurable in the serum for 26–41 weeks and had a calculated half-life of 3.8 weeks.

The effects of LS on 3BNC117 were more modest than those on 10-1074 and were consistent with a shorter half-life (2.6 versus 3.8 weeks), a smaller increase in half-life (2- versus 3.8-fold) and a lower initial serum concentration (Supplementary Table 2). These observations are also entirely in accordance with the observation that native 3BNC117 has a shorter half-life than 10-1074 in humans (17 versus 24 d)^22,23^. This difference in pharmacokinetics notwithstanding, a serum concentration of 2.68 μg/ml for either LS-modified bNAb derivative was calculated to protect 99% of the macaque recipients.

We tested combination immunoprophylaxis via the s.c. route, employing the 3BNC117-LS plus 10-1074-LS monoclonal antibodies, which target different gp120 epitopes, to model potential exposure to genetically diverse and/or resistant HIV-1 strains. Unexpectedly, four of six of these monkeys developed high titers of anti-3BNC117-LS antibodies within 5–9 weeks of administration, which resulted in the rapid elimination of this monoclonal antibody. This loss of the 3BNC117-LS component of the bNAb combination could be viewed as the biological equivalent of the previously reported emergence of viral variants resistant to an administered anti-HIV-1 monoclonal antibody^20,23,26,27,29,30^. Nonetheless, the inclusion of 10-1074-LS in the administered bNAb combination resulted in the maintenance of neutralizing activity in serum for several months, prevented the establishment of a virus infection for an additional 6–14 weeks and was solely responsible for the observed protection (median, 20 weeks) in this cohort of SHIV_AD8-EO_-challenged macaques.

Development of anti-antibodies against the administered human bNAbs in macaques was not unexpected. In fact, bNAb concentrations and neutralization titers invariably declined in 15 of the 24 monkeys infused with 3BNC117-LS and/or 10-1074-LS monoclonal antibodies when anti-bNAb titers exceeded a titer of 1:1,000 (Supplementary Fig. 3a–d). Although the generation of such antibodies will always remain a problem in the context of monkey recipients of human monoclonal antibodies, it is highly unlikely that a cross-species immune response of this frequency and potency and the attending rapid clearance of administered bNAbs will occur in humans. In this regard, two recent clinical studies have reported that VRC01 administered to healthy human adults failed to elicit detectable anti-antibodies^24,25^, and 3BNC117 and 10-1074 showed stable pharmacokinetics in humans after repeated dosing over a period of 6 months (data not shown).

Before the development of an effective vaccine against hepatitis A virus, pre-exposure immunoprophylaxis through administering immunoglobulin intramuscularly was common practice and conferred protection for 3–5 months^31^. In the absence of an effective HIV vaccine or the development of one in the immediate future, it is not unreasonable to contemplate a similar use of crystallizable fragment domain–modified anti-HIV monoclonal antibodies, such as those described in this report, which, in the case of 10-1074-LS bNAb, conferred protection in macaques against SHIV_AD8_-_EO_ for more than 6 months.

Although this study reports a promising preclinical result relevant to HIV-1 prevention, it must be remembered that to be clinically effective for immunoprophylaxis, bNAbs must be capable of preventing the acquisition of genetically diverse populations of the virus, not a molecularly cloned SHIV expressing a single envelope protein. The planned extension of the LS-modified bNAb results reported here to humans in a phase 1 clinical trial to evaluate the pharmacokinetics of 3BNC117-LS administered to groups of individuals with and without HIV infections thus represents the next step in this process (http://www.clinicaltrials.gov/; NCT03254277).

## Methods

### Rhesus monkeys

Thirty rhesus macaques (*Macaca mulatta*) of Indian genetic origin, 2–4 years of age, were housed and cared for in accordance with Guide for Care and Use of Laboratory Animals Report no. NIH 82-53 (Department of Health and Human Services, Bethesda, Maryland, 1985) in a biosafety level 2 NIH facility. All animal procedures and experiments were performed according to protocols approved by the Institutional Animal Care and Use Committee of National Institute of Allergy and Infectious Disease, NIH. Monkeys were not randomized, and the data were not collected in a blinded manner. The macaques used in this study did not express the MHC class I Mamu-A*01, Mamu-B*08 and Mamu-B*17 alleles. No monkeys were excluded from the analysis. Nine of the twelve control monkeys and recipients of the native 3BNC117 (*n* = 6) and 10-1074 (*n* = 6) bNAbs were reported in a previous study^12^. Blood was drawn regularly to monitor viral infection, passively transferred monoclonal antibody concentrations and serum neutralizing activity.

### Monoclonal antibodies

The 3BNC117 monoclonal antibody is a recombinant, fully human IgG1 λ antibody recognizing the CD4-binding site on the HIV-1 gp120 envelope^5^. This antibody was cloned from an HIV-1-infected viremic controller in the International HIV Controller Study^5,32^, and the mutations encoding the LS substitution were introduced to the heavy chain–encoding gene of 3BNC117 bNAb through site-directed mutagenesis. Plasmids encoding the heavy and light chain genes were transiently cotransfected and expressed in Chinese hamster ovary cells (clone 5D5-5C10), and supernatant was purified using standard methods. The resulting purified monoclonal antibody, designated 3BNC117-LS, was used for i.v. and s.c. injections of macaques. 10-1074 is a recombinant, fully human IgG1 λ monoclonal antibody recognizing the base of the gp120 V3 loop and surrounding glycans on the HIV-1 envelope protein^8^. The 10-1074 monoclonal antibody was cloned from an African donor (patient 10) infected with an HIV-1 clade A virus^33^. LS-encoding mutations were introduced into the heavy chain–encoding gene of 10-1074, expressed in Chinese hamster ovary cells (clone 3G4), and the purified bNAb, designated 10-1074-LS, was used in the study. A single dose (20 mg per kg body weight) of each monoclonal antibody was infused i.v. to individual monkeys.

To model immunoprophylaxis via the more clinically relevant s.c. route, a bNAb combination, which included both 3BNC117-LS and 10-1074-LS monoclonal antibodies (7.5 mg per kg body weight of each), in a total volume of 1 ml, was administered s.c. into the medial inner thigh of individual animals using a 1-inch, 25-gauge needle.

### Virus challenge

The origin and preparation of the tissue-culture-derived SHIV_AD8-EO_ stock has been previously described^34^; the infectivity of virus stock was titrated on peripheral blood mononuclear cells (PBMCs) from rhesus macaques. SHIV_AD8-EO_ is an molecularly cloned derivative of SHIV_AD8_ that is R5 tropic and possesses multiple properties typical of pathogenic HIV-1 isolates^34–36^. It exhibits a tier 2 neutralization-sensitivity phenotype, replicates to high levels in rhesus macaque PBMCs, generates sustained levels of plasma viremia and causes irreversible depletion of CD4^+^ T cells, resulting in a symptomatic and ultimately fatal immunodeficiency associated with opportunistic infections (*Mycobacterium* sp., *Pneumocystis* sp., *Cryptosporidium* sp.) in infected monkeys. All animals were inoculated through the i.r. route with 10 TCID_50_ of SHIV_AD8-EO_ at weekly intervals until infection became established. This inoculum size was previously shown to be equivalent to 0.27 AID_50_^12^. A pediatric speculum was used to gently open the rectum, and a 1-ml suspension of virus in a tuberculin syringe was slowly infused into the rectal cavity.

### Neutralization antibody assay

The in vitro potency of each monoclonal antibody was assessed using two types of neutralization assays: (1) TZM-bl entry assay with a pseudotyped virus and (2) TZM-bl infection assay using a replication-competent virus. The pseudotyped virus expresses the SHIV_AD8-EO_ envelope antigen and the luciferase reporter gene. In the case of the replication-competent virus assay, the indinavir protease inhibitor was added to the medium (final concentration of 1 µM) to prevent a second round of viral replication. Neutralization activity was quantitated by the relative decrease in the luciferase activity compared to infection of TZM.bl cells in the absence of monoclonal antibodies. Neutralization curves were subjected to fitting through nonlinear regression using a five-parameter equation using GraphPad Prism. The antibody concentrations required to inhibit infection by 50% or 80% are reported as the IC_50_ or IC_80_, respectively.

### Measurement of 3BNC117-LS and 10-1074-LS monoclonal antibody concentrations

Serum concentrations of 3BNC117-LS and 10-1074-LS bNAbs were determined using TZM.bl neutralization assays as previously described^23^. Sera were heat-inactivated for 1 h at 56 °C, and neutralizing activity was measured against two HIV-1 strains. HIV-1 strain Q769.d22 is highly sensitive to 3BNC117 but resistant to 10-1074, whereas the X2088_c9 strain is highly sensitive to 10-1074 but resistant 3BNC117. ID_50_ values were derived using five-parameter curve fitting. Serum concentrations of 3BNC117-LS and 10-1074-LS were then calculated through multiplying the respective sera ID_50_ titers by the IC_50_ values. Murine leukemia virus (MuLV)-pseudotyped viruses were used to detect nonspecific neutralizing activity in serum, which was excluded from analyses.

### Measurement of 3BNC117-LS and 10-1074-LS neutralizing activity

The neutralization activity, present in serum samples collected from rhesus macaques infused with monoclonal antibodies, was assessed through TZM-bl assay with pseudotyped SHIV_AD8_^12,16^. The IC_50_ neutralization titer was calculated as the dilution of serum causing a 50% reduction in relative luminescence units (RLUs) relative to diluted sera from untreated animals.

### Pharmacokinetic analysis

Blood samples were collected before and weekly following administration of the bNAbs. Monoclonal antibody serum concentrations were determined as described above. Pharmacokinetic parameters were estimated by performing a non-compartmental analysis using WinNonlin 6.3.

### Anti-antibody responses

ELISAs were performed to evaluate anti-3BNC117-LS or anti-10-1074-LS antibody responses in sera collected from macaques administered these monoclonal antibodies. ELISA plates (96 well) were coated with 3BNC117-LS or 10-1074-LS monoclonal antibodies in PBS (2 µg/ml), incubated at 4 °C overnight and blocked with 5% BSA at room temperature for 1 h. Plates were washed six times with PBS-Tween (PBS-T), and serial, fivefold dilutions of serum samples were prepared in 2% BSA. The diluted serum samples were added to plates and incubated at room temperature for 1 h, followed by six washes of each plate with PBS-Tween. Horseradish peroxidase (HRP)-conjugated Fcγ-specific anti–human IgG, (Jackson ImmunoResearch Labs) was added for 1 h at room temperature, followed by six washes of each plate with PBS-T. Tetramethylbenzidine (TMB)–HRP substrate was added to each well, and the absorbance developed after adding 0.5 M H_2_SO_4_ was measured at 450 nm on a EMax Plus Microplate Reader using Softmax Pro 6 software (Molecular Devices, CA, US). Blank wells containing assay diluent were used to determine the background signal; mean optical density (OD) values exceeding fivefold (ranging from 0.26 to 0.29) that of blank wells from each plate were used as cutoff. The dilution of each sample above the cutoff value was then calculated. The log values of these dilutions were reported as the final endpoint anti-bNAb titers.

### Plasma viral RNA quantification

Plasma viral RNA levels were determined through modified two-step quantitative reverse transcription using Applied Biosystems PCR (ABI Prism 7900HT). Experimental samples were analyzed in parallel with a simian immunodeficiency virus (SIV) gag RNA standard; the lower limit of detection using this assay was 100 copies/ml.

### Cell culture

HEK293T and TZM.bl cells were maintained in the DMEM media supplemented with 10% FBS, 4 mM l-glutamine and 1 × penicillin–streptomycin. Cells were passaged twice a week and incubated at 37 °C, 10% CO_2_.

### Statistical analysis

Between-group comparisons were performed using the Wilcoxon rank-sum test. The relationship between per-challenge infection and antibody concentration at the time of challenge was specified by a probit regression model: P(infection at challenge) = Φ(A + B mAb), where A and B are parameters to be estimated; mAb is the actual or imputed antibody concentration at the time of the challenge; and Φ is the standard normal cumulative distribution function. Because the monoclonal antibody concentrations were missing for some challenges, a linear mixed-effects model was fitted to the repeated monoclonal antibody readouts. Empirical Bayes–predicted monoclonal antibody concentrations were used to impute values for challenges with missing monoclonal antibody readouts. ID01 was defined as the antibody concentration resulting in an estimated per-challenge probability of infection of 0.01.

Likelihood ratio tests were performed to examine whether the different groups of monoclonal antibodies (native-10-1074, 10-1074-LS, native-3BNC117, 3BNC117-LS and combination of the two LS-modified bNAbs) had different slopes and intercepts. For each group, in turn, separate models were fitted to allow for a group-specific intercept and a group-specific intercept and slope. For each group, likelihood-ratio tests supported the simpler model with a single intercept and slope for all groups (*P* > .05). Additional likelihood-ratio tests were performed to confirm that other functions (i.e., log, quadratic) of the infused monoclonal antibody did not provide a significantly better prediction. A random-effects probit model was also assessed, and the variance of the intercept term was estimated at zero.

We calculated the reduction in the per-challenge probability of infection for a given antibody concentration, as compared to that with no antibody, using the formula antibody efficacy = 1 – Φ(a + b mAb) / Φ(a), where a and b are the maximum-likelihood estimates of A and B. The nonparametric bootstrap procedure was used to form confidence intervals for ID01 and antibody efficacy. All tests are two-sided, and *P* values less than 0.05 are considered significant.

### Reporting Summary

Further information on experimental design is available in the Nature Research Reporting Summary linked to this article.

### Data availability

The data sets generated and/or analyzed in supporting the findings of this study are available in the manuscript (and Supplementary Information). All other data are available from the corresponding authors on reasonable request.

## Methods

Methods, including statements of data availability and any associated accession codes and references, are available at 10.1038/s41591-018-0001-2.

## Electronic supplementary material

Supplementary Tables and Figures Supplementary Figures 1–3 and Supplementary Tables 1–3

Reporting Summary
