# Supplementary material for: A single injection of crystallizable fragment domain–modified antibodies elicits durable protection from SHIV infection
Source: Nat Med. 2018 Apr 16;24(5):610–6. doi: 10.1038/s41591-018-0001-2 (PMC5989326; doi:10.1038/s41591-018-0001-2)
Supplement: Supplementary file 1 — Supplementary Figures 1–3 and Supplementary Tables 1–3 [file 41591_2018_1_MOESM1_ESM.pdf]

nature medicine

SUPPLEMENTARY INFORMATION
https://doi.org/10.1038/s41591-018-0001-2

In the format provided by the authors and unedited.

OPEN

A single injection of crystallizable fragment domain-modified antibodies elicits durable protection from SHIV infection

Rajeev Gautam[1], Yoshiaki Nishimura[1], Natalie Gaughan[1], Anna Gazumyan[2], Till Schoofs[2], Alicia Buckler-White[1], Michael S. Seaman[3], Bruce J. Swihart[4], Dean A. Follmann[4], Michel C. Nussenzweig[2,5]* and Malcolm A. Martin[1]*

[1]Laboratory of Molecular Microbiology, National Institute of Allergy and Infectious Diseases, National Institutes of Health, Bethesda, MD, USA. [2]Laboratory of Molecular Immunology, Rockefeller University, New York, NY, USA. [3]Center for Virology and Vaccine Research, Beth Israel Deaconess Medical Center, Boston, MA, USA. [4]Biostatistics Research Branch, Division of Clinical Research, National Institute of Allergy and Infectious Diseases, National Institutes of Health, Bethesda, MD, USA. [5]Howard Hughes Medical Institute, Rockefeller University, New York, NY, USA. *e-mail: nussen@rockefeller.edu; malm@nih.gov

© 2018 Nature America Inc., part of Springer Nature. All rights reserved.

SUPPLEMENTARY FIGURE LEGENDS

Figure S1. Serum neutralizing antibody titers in rhesus macaques infused with the Fc modified 3BNC117-LS or 10-1074-LS mAb intravenously. Serum IC_{50} titers of the indicated mAbs were determined longitudinally against the challenge pseudotyped $SHIV_{AD8\text{-}EO}$ using the TZM-bl cell assay. The IC_{50} titers are color coded: 1:21-99 as tan; 1:100-999 as yellow and ≥ 1:1000 as orange. The neutralization assays were repeated twice.

Figure S2. Serum neutralizing antibody titers in rhesus macaques subcutaneously administered a combination of Fc modified 3BNC117-LS and 10-1074-LS mAbs. Serum IC_{50} titers of the indicated mAbs were determined longitudinally against the challenge pseudotyped $SHIV_{AD8\text{-}EO}$ using the TZM-bl cell assay. The IC_{50} titers are color coded: 1:21-99 as tan; 1:100-999 as yellow and ≥ 1:1000 as orange. The neutralization assays were repeated twice.

Figure S3. Elicitation of anti-mAb immune responses in serum of rhesus macaques receiving Fc modified anti-HIV-1 human mAbs. a, b, Sera samples were tested longitudinally for development of anti-3BNC117-LS (n = 6) or anti-10-1074-LS (n = 6) antibody responses, respectively, following a single intravenous infusion of each Fc modified mAb. **c, d,** anti-3BNC117-LS and anti-10-1074-LS antibody responses were measured longitudinally following a single subcutaneous administration of a of 3BNC117-LS plus 10-1074-LS bNAb mixture to rhesus macaques (n = 6). The ELISA to assess the anti-antibody responses in sera was performed twice.

Supplementary Figure 1. Serum neutralizing antibody titers in rhesus macaques infused with the Fc modified 3BNC117-LS or 10-1074-LS mAbs intravenously. Serum IC_{50} titers of the indicated mAbs were determined longitudinally against the challenge pseudotyped $SHIV_{AD8-EO}$ using the TZM-bl cell assay. The IC_{50} titers are color coded: 1:21-99 as gray; 1:100-999 as yellow and ≥ 1:1000 as orange. The neutralization assays were repeated twice.

	3BNC117-LS						10-1074-LS					
**Wks**	**DF6Z**	**DFH3**	**DFM1**	**DFN6**	**DFP9**	**DG43**	**DF3M**	**DF9V**	**DFB0**	**DFC6**	**DFL4**	**DFL5**
1	2282	5745	2747	2328	2845	1969	8844	8009	9822	8079	20037	10836
2	2023	3615	2087	1436	2053	1638	5231	7150	6607	8228	19589	7228
3	1605	2622	1362	982	1152	1161	3334	4232	5594	7829	13884	9093
4	1908	1935	1588	976	849	660	2502	4635	4909	7593	11865	7670
6	430	39	434	297	313	237	2073	2169	2508	1895	2693	2207
8	292	<20	341	183	268	173	1270	1245	1523	1394	2025	1738
10	235		292	82	238	170	1180	1698	1101	818	1913	1311
12	211		263	81	129	83	1124	1053	749	633	1104	659
14	95		88	27	33	46	966	704	562	264	756	341
16	67		57	26	25	40	620	412	280	189	501	234
20	<20		<20	<20	<20	<20	333	266	216	90	414	134
24							251	136	32		220	55
28							80	90	<20	41	68	<20
32							<20	82	<20	<20	<20	<20
36								68				
40								<20				

Supplementary Figure 2. Serum neutralizing antibody titers in rhesus macaques subcutaneously administered a combination of Fc modified 3BNC117-LS and 10-1074-LS mAbs. Serum IC_{50} titers of the indicated mAbs were determined longitudinally against the challenge pseudotyped $SHIV_{AD8-EO}$ using the TZM-bl cell assay. The IC_{50} titers are color coded: 1:21-99 as gray; 1:100-999 as yellow and ≥ 1:1000 as orange. The neutralization assays were repeated twice.

Wks	DFD7	DFJ4	DFM6	DFN9	DFX9	DGGV
1	5143	3561	4353	2535	2730	3407
2	3457	3272	3028	2051	2266	2133
3	1871	1989	1979	1411	1841	1642
4	1836	1467	1927	1326	1311	1574
6	917	1010	<20	896	1149	894
8	865	535		630	740	518
10	742	439		439	414	463
12	395	170		253	315	200
14	374	153		206	254	170
16	350	150		191	207	177
20	235	119		170	<20	117
24	98	<20		56		40
28	<20			<20		<20

Supplementary Figure 3. Elicitation of anti-mAb immune responses in serum of rhesus macaques receiving Fc modified anti-HIV-1 human mAbs. a, b, Sera samples were tested longitudinally for development of anti-3BNC117-LS ($n = 6$) or anti-10-1074-LS ($n = 6$) antibody responses, respectively, following a single intravenous infusion of each Fc modified mAb. **c, d,** anti-3BNC117-LS and anti-10-1074-LS antibody responses were measured longitudinally following a single subcutaneous administration of a of 3BNC117-LS plus 10-1074-LS bNAb mixture to rhesus macaques ($n = 6$). The ELISA to assess the anti-antibody responses in sera was performed twice.

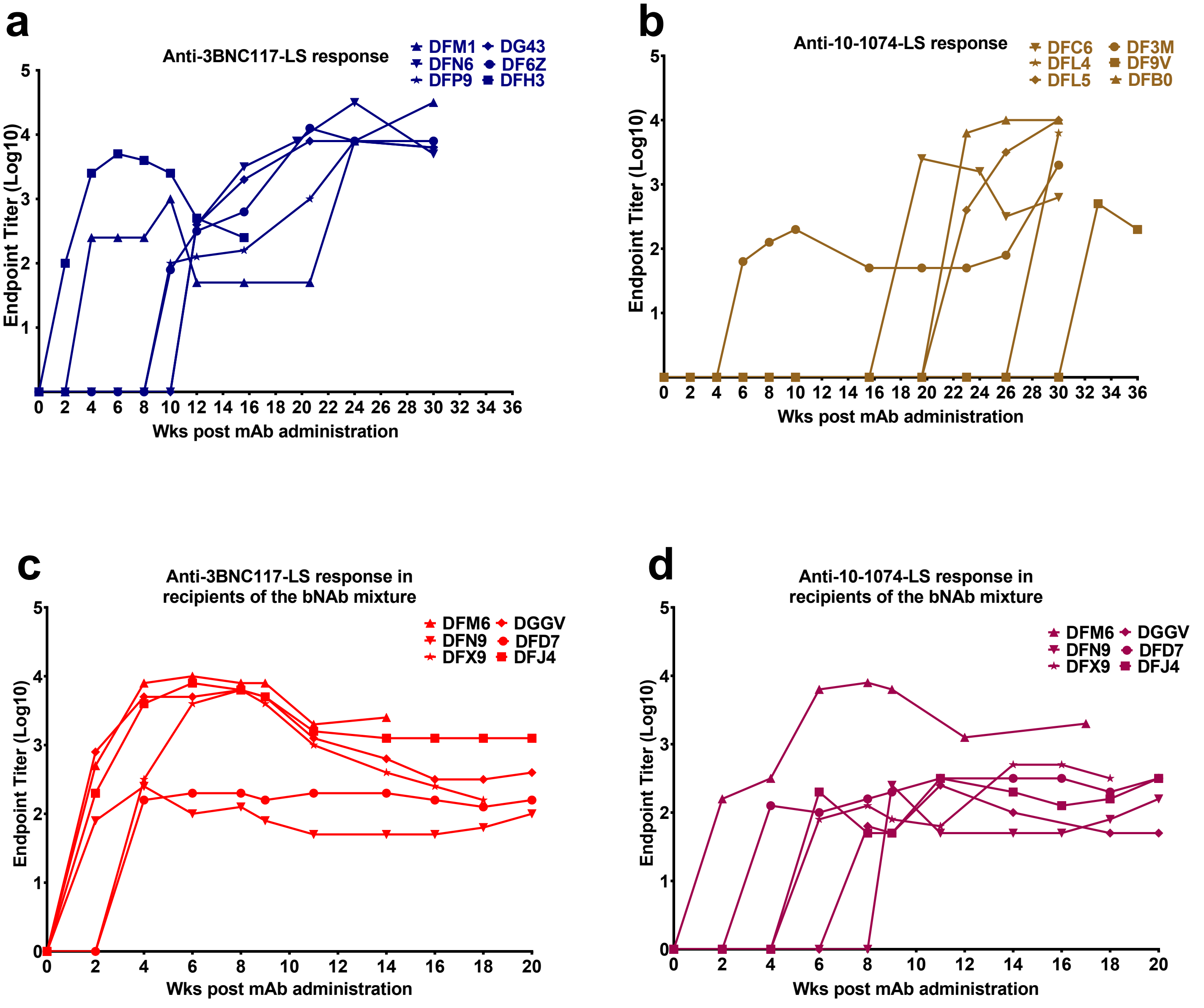

Supplementary Table 1. 3BNC117-LS or 10-1074-LS antibody concentrations in serum of macaques after a single intravenous infusion of indicated mAbs

	3BNC117-LS conc (µg/ml)					
**Wks**	**DF6Z**	**DFH3**	**DFM1**	**DFN6**	**DFP9**	**DG43**
**1**	46.02	76.18	38.39	40.19	33.52	22.97
**3**	16.13	29.47	19.75	14.08	20.35	9.17
**5**	10.13	5.19	10.85	5.48	9.07	4.13
**7**	11.02	ND	10.52	5.06	8.84	1.89
**9**	6.01		6.59	2.00	4.32	1.09
**11**	2.69		3.54	0.90	2.64	0.75
**14**	1.31		2.00	0.46	1.15	0.51
**17**	0.82		0.78	ND	ND	0.34
**18**	1.09					ND
**20**			ND			
**22**	ND					

ND non-detectable

	10-1074-LS conc (µg/ml)					
**Wks**	**DF3M**	**DF9V**	**DFB0**	**DFC6**	**DFL4**	**DFL5**
**1**	176.87	110.57	297.84	92.77	112.75	115.19
**3**	63.16	58.55	78.61	46.33	52.10	58.91
**5**	54.07	47.37	34.21	31.47	23.05	34.35
**7**	42.58	41.75	26.06	29.27	16.58	20.99
**9**	34.11	23.21	14.39	9.04	12.01	11.68
**11**	19.11	14.92	14.44	5.59	10.90	11.21
**14**	13.64	9.09	8.56	3.41	6.71	5.89
**17**	5.70	6.39	4.48	1.66	3.86	2.69
**20**	6.49	5.25	2.83	1.44	2.59	1.37
**22**	4.14	3.92	2.15	0.65	2.62	1.04
**24**	2.32	2.70	1.04	0.24	2.55	0.67
**26**	2.08	1.66	0.14	0.29	1.77	0.57
**28**	1.76	1.70	ND	0.32	1.06	ND
**31**	0.73	1.78		0.12	0.08	
**33**		0.68		ND	ND	
**37**	ND	0.49				
**38**		0.34				
**41**		0.22				
**43**		ND				

ND non-detectable

Supplementary Table 2 . Half-lives of native 3BNC117, native10-1074 and LS-derivative bNAbs, administered intravenously in macaques

**native 3BNC117**		**3BNC117-LS**		**native 10-1074**		**10-1074-LS**	
**Monkey**	**Half-life (weeks)**	**Monkey**	**Half-life (weeks)**	**Monkey**	**Half-life (weeks)**	**Monkey**	**Half-life (weeks)**
DFGP	1.64	DF6Z	3.24	DF60	0.34	DF3M	4.40
DFIP	1.30	DFH3	1.46	DF80	0.45	DF9V	4.90
DFKV	0.71	DFM1	3.00	DFAM	1.68	DFB0	3.16
M57	1.61	DFN6	2.16	DFCP	0.55	DFC6	3.01
MMK	1.24	DFP9	2.68	DFDP	3.25	DFL4	4.19
MRF	1.65	DG43	2.91	DFFN	1.54	DFL5	3.37
Median (weeks)	**1.45**	Median (weeks)	**2.79**	Median (weeks)	**1.05**	Median (weeks)	**3.78**
Mean (weeks)	**1.37**	Mean (weeks)	**2.58**	Mean (weeks)	**1.30**	Mean (weeks)	**3.84**

The half-life was estimated by WinNonlin 6.3 software using non-compartmental analysis (NCA) of the serum concentrations of 3BNC117-LS and 10-1074-LS mAbs.

Supplementary Table 3. 3BNC117-LS and 10-1074-LS antibody concentrations in serum of macaques after a single subcutaneous administration of LS-mAb mixture

	3BNC117-LS conc (μg/ml)					
**Wks**	**DFD7**	**DFJ4**	**DFM6**	**DFN9**	**DFX9**	**DGGV**
**1**	9.55	11.45	11.12	4.20	7.15	8.13
**3**	4.77	3.74	2.31	1.90	4.00	2.72
**5**	4.84	ND	ND	2.20	2.27	1.98
**7**	2.13			0.48	ND	ND
**9**	1.67			ND		
**12**	1.24					
**14**	0.78					
**16**	0.84					
**18**	ND					

ND non-detectable

	10-1074-LS conc (μg/ml)					
**Wks**	**DFD7**	**DFJ4**	**DFM6**	**DFN9**	**DFX9**	**DGGV**
**1**	35.65	45.86	49.05	34.71	23.14	34.00
**3**	19.53	21.43	21.78	14.67	11.44	27.09
**5**	11.45	9.05	4.31	12.04	8.02	7.50
**7**	12.20	4.86	ND	5.13	8.25	3.90
**9**	7.68	4.49		3.83	5.91	2.69
**12**	4.54	2.58		2.86	2.66	2.77
**14**	3.51	1.08		2.63	1.85	1.32
**16**	4.58	1.45		2.24	2.058	1.07
**18**	2.85	1.00		1.22	1.284	0.89
**20**	2.02	0.58		0.94	ND	0.88
**23**	1.26	0.21		0.73		0.26
**24**	1.209	0.174		0.42		0.195
**26**	0.669	ND		ND		ND
**28**	ND					

ND non-detectable
