# Supplementary material for: A single injection of crystallizable fragment domain–modified antibodies elicits durable protection from SHIV infection
Source: Nat Med. 2018 Apr 16;24(5):610–6. doi: 10.1038/s41591-018-0001-2 (PMC5989326; doi:10.1038/s41591-018-0001-2)
Supplement: Supplementary file 2 — Reporting Summary [file 41591_2018_1_MOESM2_ESM.pdf]

nature research

Corresponding author(s): NMED-A88614A

Life Sciences Reporting Summary

Nature Research wishes to improve the reproducibility of the work that we publish. This form is intended for publication with all accepted life science papers and provides structure for consistency and transparency in reporting. Every life science submission will use this form; some list items might not apply to an individual manuscript, but all fields must be completed for clarity.

For further information on the points included in this form, see Reporting Life Sciences Research. For further information on Nature Research policies, including our data availability policy, see Authors & Referees and the Editorial Policy Checklist.

Please do not complete any field with "not applicable" or n/a. Refer to the help text for what text to use if an item is not relevant to your study. For final submission: please carefully check your responses for accuracy; you will not be able to make changes later.

Experimental design

1. Sample size

Describe how sample size was determined.

In total, 30 rhesus macaques were used for this study. 12 animals were challenged with SHIVAD8 without any prior MAb infusion and served as controls, nine of these twelve control monkeys were reported in a previous study {Gautam, R., et al. Nature 533, 105-109 (2016)}. 18 animals (3 groups of 6 animals) were infused with indicated MAbs followed by intrarectal challenges with SHIVAD8. No statistical methods were used to predetermine sample sizes.

2. Data exclusions

Describe any data exclusions.

No data excluded from the study.

3. Replication

Describe the measures taken to verify the reproducibility of the experimental findings.

Yes, we have a rigorous process of doing all in vitro assays at least twice to maintain reproducibility. All the attempts at replication were successful and the results were reliably reproduced.

4. Randomization

Describe how samples/organisms/participants were allocated into experimental groups.

No method of randomization was used to determine how animals were allocated to experimental groups, which was determined by genotype. Macaques carrying protective MHC class 1 alleles (Mamu A*01, B*O8, B*17) were excluded from this study.

5. Blinding

Describe whether the investigators were blinded to group allocation during data collection and/or analysis.

The data collection was not blinded. Blinding was not possible as the investigators were also conducting the experiments and had to be aware of controls and treated groups.

Note: all in vivo studies must report how sample size was determined and whether blinding and randomization were used.

6. Statistical parameters

For all figures and tables that use statistical methods, confirm that the following items are present in relevant figure legends (or in the Methods section if additional space is needed).

n/a	Confirmed	
☐	☒	The exact sample size (n) for each experimental group/condition, given as a discrete number and unit of measurement (animals, litters, cultures, etc.)
☐	☒	A description of how samples were collected, noting whether measurements were taken from distinct samples or whether the same sample was measured repeatedly
☐	☒	A statement indicating how many times each experiment was replicated
☐	☒	The statistical test(s) used and whether they are one- or two-sided<br>*Only common tests should be described solely by name; describe more complex techniques in the Methods section.*
☐	☒	A description of any assumptions or corrections, such as an adjustment for multiple comparisons
☐	☒	Test values indicating whether an effect is present<br>*Provide confidence intervals or give results of significance tests (e.g. P values) as exact values whenever appropriate and with effect sizes noted.*
☐	☒	A clear description of statistics including central tendency (e.g. median, mean) and variation (e.g. standard deviation, interquartile range)
☐	☒	Clearly defined error bars in all relevant figure captions (with explicit mention of central tendency and variation)

See the web collection on statistics for biologists for further resources and guidance.

Software

Policy information about availability of computer code

7. Software

Describe the software used to analyze the data in this study.	Prism, Microsoft Excel

For manuscripts utilizing custom algorithms or software that are central to the paper but not yet described in the published literature, software must be made available to editors and reviewers upon request. We strongly encourage code deposition in a community repository (e.g. GitHub). *Nature Methods* guidance for providing algorithms and software for publication provides further information on this topic.

Materials and reagents

Policy information about availability of materials

8. Materials availability

Indicate whether there are restrictions on availability of unique materials or if these materials are only available for distribution by a third party.	No restriction

9. Antibodies

Describe the antibodies used and how they were validated for use in the system under study (i.e. assay and species).	Human mAbs were used for in vitro and in vivo experiments. Neutralizing potencies of mAbs were assessed in vitro against panels of HIV-1 isolates or SHIVAD8 using TZMbl (human cells) entry assay.

10. Eukaryotic cell lines

a. State the source of each eukaryotic cell line used.	TZM-bl and HEK293 T cells were obtained cells from NIH AIDS Research and Reference Reagent Program.
b. Describe the method of cell line authentication used.	None of the cell lines have been authenticated.
c. Report whether the cell lines were tested for mycoplasma contamination.	Not tested
d. If any of the cell lines used are listed in the database of commonly misidentified cell lines maintained by ICLAC, provide a scientific rationale for their use.	No commonly misidentified cell lines were used.

Animals and human research participants

Policy information about studies involving animals; when reporting animal research, follow the ARRIVE guidelines

11. Description of research animals

Provide all relevant details on animals and/or animal-derived materials used in the study.	Rhesus macaques (Macaca mulatta) of Indian genetic origin, male and female, 2 to 4 years of age, negative for MHC alleles associated with SIV control (Mamu A*01, B*08, and B*17).

Policy information about studies involving human research participants

12. Description of human research participants

Describe the covariate-relevant population characteristics of the human research participants.	No human subjects were used.
